# A Short Mindfulness Retreat for Students to Reduce Stress and Promote Self-Compassion: Pilot Randomised Controlled Trial Exploring Both an Indoor and a Natural Outdoor Retreat Setting

**DOI:** 10.3390/healthcare9070910

**Published:** 2021-07-18

**Authors:** Dorthe Djernis, Mia S. O’Toole, Lone O. Fjorback, Helle Svenningsen, Mimi Y. Mehlsen, Ulrika K. Stigsdotter, Jesper Dahlgaard

**Affiliations:** 1 Department of Geosciences and Natural Resource Management, University of Copenhagen, DK-1958 Frederiksberg, Denmark; Uks@ign.ku.dk; 2 Department of Psychology and Behavioural Sciences, Aarhus University, DK-8000 Aarhus, Denmark; mia@psy.au.dk (M.S.O.); mimim@psy.au.dk (M.Y.M.); 3 Department of Clinical Medicine, Aarhus University, DK-8200 Aarhus, Denmark; Lone.overby.fjorback@clin.au.dk (L.O.F.); jesd@via.dk (J.D.); 4 Program for Mind and Body in Mental Health, Research Centre for Health and Welfare Technology, VIA University College, DK-8200 Aarhus, Denmark; Hesv@via.dk

**Keywords:** meditation, nature-based mindfulness, stress reduction, self-compassion, retreat

## Abstract

Here, we developed and examined a new way of disseminating mindfulness in nature to people without meditation experience, based on the finding that mindfulness conducted in natural settings may have added benefits. We evaluated a 5-day residential programme aiming to reduce stress and improve mental health outcomes. We compared an indoor and an outdoor version of the programme to a control group in a pilot randomised controlled trial (RCT). Sixty Danish university students experiencing moderate to high levels of stress were randomised into a residential mindfulness programme indoors (*n* = 20), in nature (*n* = 22), or a control group (*n* = 18). Participants completed the Perceived Stress Scale and the Self-Compassion Scale (primary outcomes) along with additional secondary outcome measures at the start and end of the program and 3 months after. Stress was decreased with small to medium effect sizes post-intervention, although not statistically significant. Self-compassion increased post-intervention, but effect sizes were small and not significant. At follow-up, changes in stress were not significant, however self-compassion increased for both interventions with medium-sized effects. For the intervention groups, medium- to large-sized positive effects on trait mindfulness after a behavioural task were found post-intervention, and small- to medium-sized effects in self-reported mindfulness were seen at follow-up. Connectedness to Nature was the only outcome measure with an incremental effect in nature, exceeding the control with a medium-sized effect at follow-up. All participants in the nature arm completed the intervention, and so did 97% of the participants in all three arms. Overall, the results encourage the conduct of a larger-scale RCT, but only after adjusting some elements of the programme to better fit and take advantage of the potential benefits of the natural environment.

## 1. Introduction

Stress and related conditions such as depression and anxiety are prevalent in modern societies, particularly among late adolescents and students [1,2,3,4,5]. This underlines the need for evidence-based programmes and interventions to prevent and reduce stress and related conditions.

Mindfulness programmes developed for different clinical groups and purposes have been shown to reduce stress. Examples include the 8-week Mindfulness-Based Stress Reduction (MBSR) programme [6] and the Mindfulness-Based Cognitive Therapy (MBCT) programme [7]. They have proven effective in reducing stress and enhancing self-compassion [8,9]. Gilbert defines compassion as ‘a sensitivity to suffering in self and others, with a commitment to try to alleviate and prevent it’ [10] (p. 19) and claims that compassion is key to self-regulation and stress reduction [11]. Such attitudes of warmth and compassion are believed to allow awareness of present-moment experiences and space for self-nourishment when experiences are painful [12].

Substantial empirical evidence has been gathered about mindfulness programmes such as the MBSR and MBCT [13]. However, average dropout rates in these programmes vary between 16% [14] and 29% [15]. In order to reduce dropout, alternative formats for providing the curriculum should be explored. Examples of existing alternative formats include brief (e.g., 4 weeks) mindfulness interventions [16], online formats [17] and short, intensive residential programmes (i.e., retreats). A systematic review and meta-analysis [18] shows that only meditation retreats with non-novice meditators have been investigated; this leaves a gap in research concerning meditation retreats aimed at novice meditators.

Another alternative to traditional mindfulness programmes is mindfulness training conducted in natural outdoor settings. In itself, exposure to nature has been proven to reduce stress, enhance self-compassion and restore attention [8,19]. Stress reduction in natural settings may be mediated by restored attention [20]. Kaplan and Kaplan’s attention restoration theory (ART) [21] proposes that depleted attention is more easily restored in natural environments because they facilitate effortless scanning attention and offer stimuli patterns that are sufficiently interesting to call for exploration and curiosity [22]. ART is supported by a wide range of studies, including a meta-analysis of 42 studies showing that working memory, cognitive flexibility, and attentional control are improved after exposure to nature, with low to moderate effect sizes [23].

Kaplan suggests that the processes of attention restoration in natural environments are similar in some respects to the processes of meditation [24]. In natural environments, attention restoration can happen effortlessly and can be experienced without prior training. In meditation, attention restoration happens when attention is brought, for instance, to the breath or to sounds; for experienced meditators, this is believed to occur somewhat effortlessly. It is possible that people with little or no meditation experience, who are dependent on effortful (top–down) attention regulation during meditation [25,26], may feel supported by the natural environment, where the surroundings themselves assist a more effortless restoration of attention [24].

A recent meta-analysis by Djernis et al. [27] examined 25 studies on nature-based mindfulness. The analysis showed that meditation in natural settings enhances positive effects on psychological, physical, and social outcomes compared with indoor meditation, corresponding to a medium effect size (g = 0.54). With particular relevance for our study, two studies investigated indoor and outdoor mindfulness programmes with weekly attendance (i.e., MBSR and MBCT) [28,29]. Both programmes had significant effects on physical and psychological outcomes. After a 3-month intervention, Willert et al. [29] found a significant medium effect size on the Perceived Stress Scale (PSS), both when the programme was conducted indoors and when it was outdoors in a natural setting. With another manual-based meditation programme conducted in a natural outdoor setting, Lymeus et al. [30] showed an increase in attention performance. For participants with concentration challenges, the programme outperformed a traditional indoor mindfulness programme [31]. Combined, these studies and a recent one by Choe et al. [32] point to a potential added benefit of practising mindfulness in natural outdoor settings, especially for individuals with little or no meditation experience. In addition, they reveal a gap in research concerning randomised controlled studies of manual-based stand-alone mindfulness programmes conducted in natural settings.

To broaden the spectrum of interventions for stressed individuals with little or no meditation experience and to reduce dropout compared with manual-based programmes with weekly attendance, our pilot randomised controlled trial (RCT) was set up to investigate the effects of two alternative versions of traditional programmes with weekly attendance: a 5-day indoor residential mindfulness programme based on the 8-week MBSR programme, and the MBSR programme conducted in a natural outdoor setting.

The primary hypotheses were (1) that the 5-day indoor programme would prove to be more effective than a passive control condition, and (2) that the outdoor programme in a natural setting would show incremental effects compared with the 5-day indoor residential programme. Accordingly, the first objective of this pilot study was to determine the effectiveness of the 5-day residential indoor mindfulness programme compared with a control group in the treatment of stress among Danish students. The second objective was to determine whether the same 5-day residential programme conducted in a natural setting was as effective as or even more effective than the same programme conducted indoors. With these objectives, we hoped to shed light on both the potential effectiveness and the clinical relevance of the 5-day programme.

## 2. Materials and Methods

### 2.1. Trial Design

The trial was designed as an explorative three-armed parallel RCT pilot study to assess the potential effectiveness of a 5-day residential mindfulness intervention conducted indoors and possible incremental effects when the programme was conducted in a natural setting. Due to slow recruitment during the first year, the control group was postponed to year 2. Recruitment took place for two successive years (2016 and 2017). The study was approved by the Regional Science Ethical Committees (registration number H-15010925) and the Danish Data Protection Agency, and preregistered at ClinicalTrials.gov (NCT02867657).

### 2.2. Participants and Procedure

With an expected between-group difference from pre- to post-treatment with an effect size of d = 0.5 [33] and statistical power of 90% with an α of 0.05, we needed 54 participants. Students were recruited from universities and university colleges by means of intranet announcements, flyers, and referrals by university counsellors. Information about the project was made available on a project website between April and August (in both 2016 and 2017). Students were offered the opportunity to complete an online screening questionnaire containing enquiries regarding stress symptoms and the inclusion criteria. After initial screening, the students were invited to an information meeting and were individually interviewed by a psychologist with the aim of determining eligibility. The inclusion criteria were as follows:

1. Participants were active bachelor’s or master’s degree students at Danish universities or university colleges;
2. They had elevated self-reported perceived stress at the time of enrolment in the project, indicated by a PSS score of 16 or above [34];
3. They had no known psychiatric diagnosis such as severe depression, severe anxiety, adjustment disorder, post-traumatic stress disorder, personality disorder, or psychosis, and no known autism or untreated attention deficit hyperactivity disorder;
4. They had no self-reported risk of suicide or addiction to alcohol, tobacco, or drugs.

Participants completed questionnaires containing background and demographic questions and outcome measures upon arrival and before departure from the 5-day residential mindfulness retreat. Effects were evaluated both post-treatment and at follow-up. Just before the intervention, on the first day (T1), a personal link to online questionnaires was sent to the participants, who completed these questionnaires in addition to the Breath-Counting Task (BCT, see below) [35]. The same procedure was repeated just after the 5-day intervention (T2). Links to the online questionnaires and the BCT were then sent to participants 3 months after the intervention (T3). They were reminded of this by e-mail 1 week beforehand and were contacted by e-mail, and later by text message, if they did not respond or did not fill in the questionnaire. The control group followed the same schedule and procedures. The data for all three groups were collected indoors at T1 and T2. Students assigned to the outdoor mindfulness programme completed the data collection before entering the natural setting.

### 2.3. Randomisation

To ensure an equal number of students in all groups, an independent researcher made a block randomised list in Excel for the group allocation [36]. The first block contained 24 individuals (eight for each group), and the subsequent blocks included 12 (four in each group) and then six (two in each group) to ensure an equal number of students in all groups. The list was handed over to a colleague who was external to the project to ensure concealment of the randomisation list. Each time a student sent an informed consent form to the project coordinator (the first author or a delegate), the colleague was asked by text message to send the next group allocation on the list. During the recruitment period, eligible students who had signed the informed consent form were randomised in the order in which their signed forms were received. This assignment strategy was chosen to ensure a sufficient number of participants. The included students were allocated into three groups: one undertaking an indoor mindfulness retreat, one undertaking a mindfulness retreat in a natural setting, and a control group. The students were informed of their group affiliation by e-mail. No incentives were given except the treatment itself, which was provided free of charge, including accommodation and meals. The control group members were offered a 2-day mindfulness retreat in a natural setting to be conducted after the data had been collected.

### 2.4. Interventions

Kabat-Zinn’s [6] 8-week MBSR programme was adjusted to fit a 5-day residential intervention (Table 1). The groups comprised 7–15 participants. At each retreat, there were two certified MBSR teachers: one with 20 years of experience in teaching meditation retreats, the other a psychologist with 20 years of training and with experience in teaching mindfulness in natural settings. Each session of the MBSR programme was taught as in the original programme, including invitations to practise informal mindfulness between sessions and with additional morning and evening sessions of sitting meditation, yoga, and silent formal and informal mindful walks (Table 2). One primary focus was on the cultivation of compassion towards oneself and others, both in the guiding of the practices and as embodied by the teachers and staff, who attended the sessions and were available for informal conversations between sessions.

The two intervention groups not only went through the same curriculum but they also had the same teachers and staff, the same diet, and the same accommodation: they all slept outdoors in standard individual tents adjacent to the intervention area. The difference between the interventions was the setting. Two universities served as study settings. The indoor mindfulness intervention took place at a university college in August during the summer holidays, when there was no educational activity on campus. The buildings date from 2011, with large glass facades allowing extended views across a suburban area with office buildings and a car park. A room with a wooden floor was reserved for the mindfulness classes and was kept tidy during the retreat. The outdoor mindfulness intervention took place in a 14,000 square meter therapy garden belonging to the University of Copenhagen, designed to support a specific nature-based therapy programme for individuals suffering from stress-related illnesses. It contains several features, including a space for bonfires, ponds, a stream, a large greenhouse with a small kitchen, an area with wooden decking, and lush vegetation. The therapy garden is situated in an arboretum that gives the experience of a full-grown forest. At night, the outdoor temperature range for the group undertaking mindfulness sessions outdoor was 10–18 °C; the night-time temperature range was 7–17 °C for the indoor group. The outdoor temperature range during the day was 11–30 °C. The average daily rainfall was 0.13 cm, the average sunshine was 8.6 h, and the maximum wind speed was below 6 m per second.

Participants who had been randomly allocated to the control group were not restricted in terms of their activities or diet. However, they were asked not to participate in anything that differed much from their normal everyday activities and that might affect their condition, such as fasting or unusually stressful activities.

### 2.5. Primary Outcomes

The primary outcomes were self-compassion and perceived stress levels. The 12-item, short-form Self-Compassion Scale (SCS-SF) measures the extent to which individuals treat themselves with kindness and concern when faced with loss, failure, rejection, etc. [36]. It contains six subscales: self-kindness, self-judgement, common humanity, isolation, mindfulness, and overidentification. Each item on the SCS-SF is scored on a five-point Likert scale, with responses ranging from one (‘almost never’) to five (‘almost always’) to give a total sum score (range 12–60). The SCS-SF was validated in a US sample of students (n = 415) and demonstrated adequate internal consistency, with Cronbach’s α ≥ 0.86 [37]. Cronbach’s α was 0.88 for the total scale in the present study.

The 10-item PSS [34] measures the extent to which individuals globally find their lives to be unpredictable, uncontrollable and overloaded. Each item on the PSS-10 is scored on a five-point Likert scale, with responses ranging from zero (‘never’) to four (‘very often’), to give a total sum score (range 0–40). The Danish PSS-10 consensus version has been validated in a sample of 64 patients with work-related stress, with Cronbach’s α = 0.84 [38]. Cronbach’s α was 0.83 in the present study.

### 2.6. Secondary Outcomes

The 39-item Five-Facet Mindfulness Questionnaire (FFMQ) [39] measures trait mindfulness and includes the facets ‘observe’, ‘describe’, ‘non-judgement’, ‘non-reactivity’, and ‘acting with awareness’. Each item is rated on a five-point Likert scale, indicating how often each statement is true in general for the respondent, to give a total sum score (range 39–195). The Danish version used in this study was validated in a series of studies [40], and the scores showed adequate internal consistency in a healthy, randomly invited non-meditating adult community sample (n = 490, Cronbach’s α = 0.72–0.91) and in healthy university students (n = 99, all Cronbach’s α ≥ 0.78) [40]. Cronbach’s α was 0.91 for the total scale in the present study.

The 14-item Connectedness to Nature Scale (CNS) measures individuals’ trait levels of feeling emotionally connected to the natural world [41]. Each item on the CNS is scored on a five-point Likert scale, with responses ranging from one (‘strongly agree’) to five (‘strongly disagree’), to give a total sum score (range 14–70). Its validity and reliability have been tested in five studies in the US using both student and community samples, with a total of 624 individuals (Cronbach’s α = 0.72–0.84) [41]. The CNS was translated into Danish using the procedure of forward-translation and back-translation: 1) psychologists experienced in this field (the first author and a colleague) translated the original CNS into Danish; 2) professional translators translated it back into English; 3) the authors of the CNS compared the original scale with the back-translation and made sure it matched the original. Cronbach’s α was 0.85 in the present study.

The Breath-Counting Task (BCT) is a behavioural tool that measures trait mindfulness [35]. In a computer-based programme accessible online from a personal computer, the individual is instructed to press a specific button for each breath from one to eight breaths, and another button for the ninth breath. This is repeated for 15 min. Mind-wandering is believed to cause inaccurate button-pressing by the respondent. Counting accuracy (accurate presses/total presses × 100) is therefore taken to reflect the ability to stay present. The validity and reliability of the BCT have been previously documented (intraclass correlation coefficient of 0.60 in [35]; intraclass correlation coefficient of 0.48 in [42]).

### 2.7. Analytic Strategy

Mixed linear models were employed to compare groups over time on the primary (SCS and PSS) and secondary (FFMQ, CNS and BCT) outcome variables, which were all treated as continuous variables. The analyses were based on the T1 completers, and all individuals, therefore, appeared in the analyses with the number of subsequent observations completed. At the scale level, missing values were mean substituted if the respondent had answered a minimum of 50% of the items [43]. The data were hierarchically arranged into two levels, where time at level one was nested within individuals at level two. Fixed effects were specified for intercept, time, group, and a time x group interaction. All models also included a random intercept, and the slope was specified as random in analyses with more than two time points, and if it improved the model fit as evaluated by a change in the -2LL (minus twice the log likelihood) fit statistic [44].

We first investigated the effect of the 5-day indoor mindfulness intervention by testing the interaction term between time and group (i.e., indoor, control), both immediately post-intervention and at the 3-month follow-up. We benchmarked these effects with those obtained in traditional 8-week programmes. We did this to ensure that our shortened programme was feasible. Primary analyses consisted of mixed linear models investigating differences between all three groups (nature, indoor, control), both immediately post-intervention and at follow-up.

Given the relatively small sample size, we interpreted the results not simply according to *p*-values but also according to the obtained effect size. Here, a Cohen’s d larger than 0.5 has often been considered clinically relevant [45]. Effect sizes were expressed as Hedge’s g, a variant of Cohen’s d adjusted for small sample sizes, where >0.2, >0.5 and >0.8 were considered small, medium, and large effect sizes, respectively [46]. Cohen’s d was derived from the F-test calculated as d = 2*√(F/df). IBM SPSS version 25 (IBM, Chicago, IL) was used for all analyses.

## 3. Results

### 3.1. Characteristics of Participants

**Figure 1:**
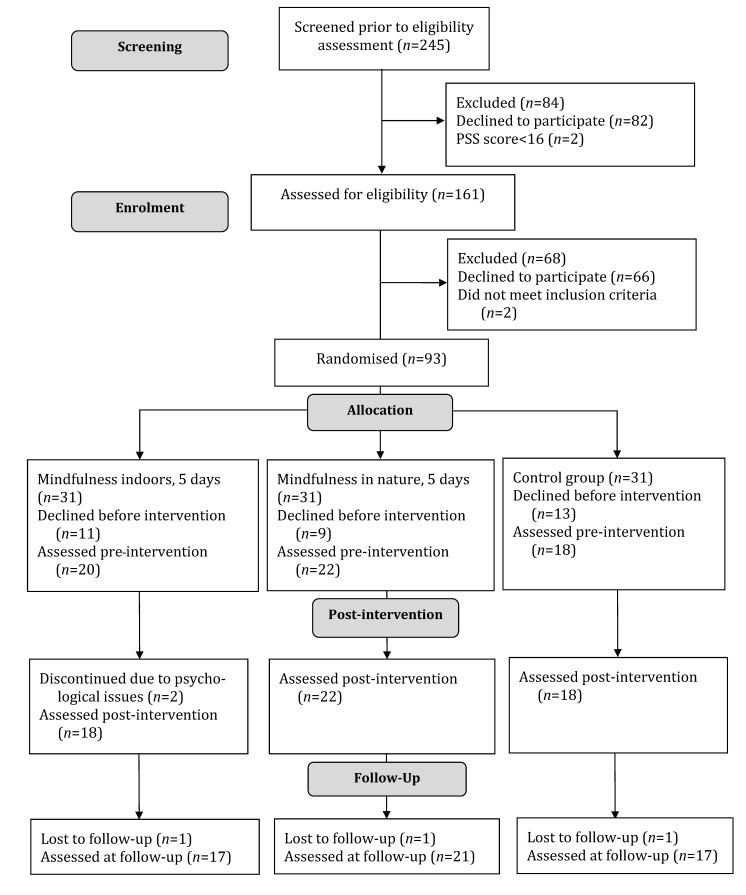
Flow chart, enrolment of students.

Between April and August in both 2016 and 2017, a total of 245 students filled in an online screening tool. Of those students, 82 chose not to proceed after the initial screening, and two had a PSS score <16 and were excluded. The remaining 161 (65.7%) were assessed for eligibility. Two were considered ineligible due to low stress levels, and 66 others declined to participate due to scheduling challenges. The remaining 93 students signed informed consent forms and were randomised into a mindfulness in nature group (n = 31), a mindfulness indoor group (n = 31), and a control group (n = 31). Before the intervention began, a further 33 students dropped out due to scheduling conflicts (Figure 1). No significant differences between the dropouts and the included participants were found with regard to age (*p* = 0.313), gender (*p* = 0.137), or PSS score (*p* = 0.279) at screening. Of the remaining 60 participants, 86.7% were female, the mean age was 30.6 years (range 21–60), and 98.3% were ethnic Danish. Of the 60 students who participated in the trial, two dropped out during treatment (3.3%), and three were lost to follow-up (5.0%).

The baseline characteristics of the three groups were well balanced with regard to age, prior mindfulness training, prior experience of nature, and outcome variables (Table 3). Although five of the eight males attended the indoor mindfulness treatment, a chi-squared test showed no group differences concerning gender (χ^2^(2) = 3.6, *p* = 0.162). The distribution of ethnicity was not calculated, as all but one of the participants were ethnic Danish.

### 3.2. Effects of the 5-Day Residential Mindfulness Interventions

**Figure 2:**
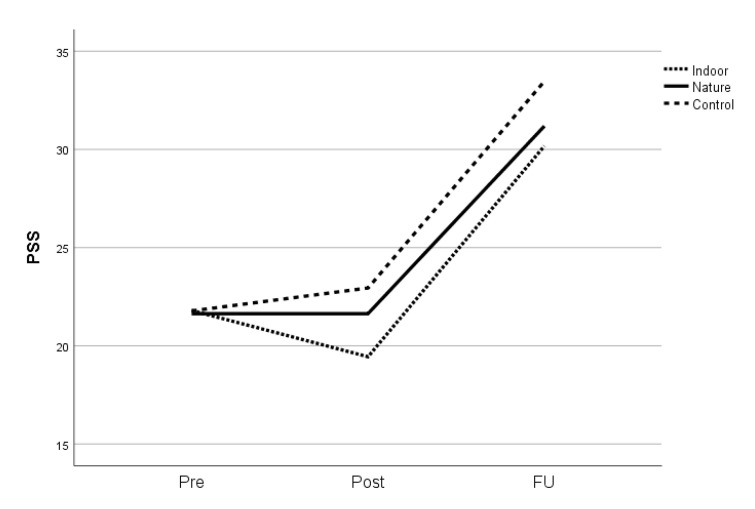
Effects on perceived stress level (PSS). Note. Pre-treatment, post-treatment, and at 3-month follow-up (FU). Dotted line: mindfulness indoors. Solid line: mindfulness in natural setting. Hatched line: control group.

**Figure 3:**
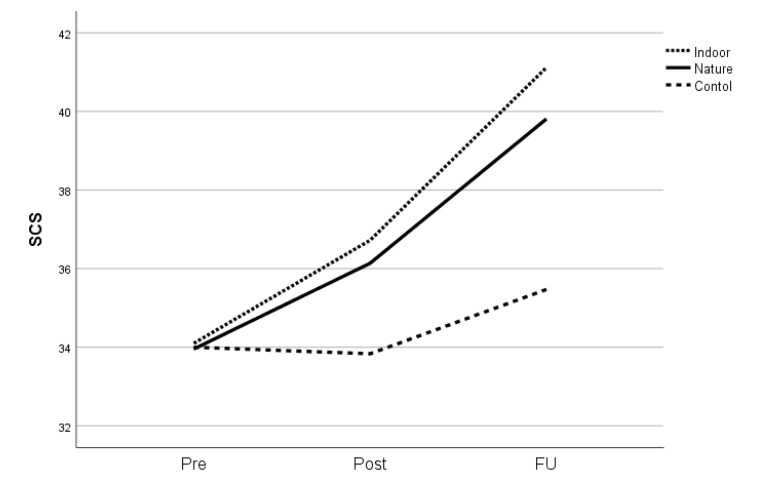
Effects on self-compassion (SCS). Note. Pre-treatment, post-treatment, and at 3-month follow-up (FU). Dotted line: mindfulness indoors. Solid line: mindfulness in natural setting. Hatched line: control group.

In order to explore the effects of our shortened mindfulness programme, we initially focused on a comparison between the indoor mindfulness intervention and the control conditions. For the two primary outcomes, there were no significant effects post-treatment when the two groups were compared (Table 4, ‘control versus indoor’). However, there was an effect size above 0.5 post-treatment for perceived stress (PSS) (Table 4, Figure 2). At follow-up, the effects of the indoor intervention compared with passive control conditions were significant for self-compassion (SCS) (F = 5.4, *p* = 0.023, g = 0.54) (Figure 3). For the secondary outcomes, only mindfulness measured by BCT showed significant effects post-treatment in the indoor mindfulness group compared with passive control conditions (F = 7.6, *p* = 0.009, g = 0.90), whereas the effect on FFMQ post-treatment may be clinically relevant (F = 2.3, *p* = 0.136, g = 0.50). At follow-up, mindfulness measured by FFMQ was significant (F = 9.8, *p* = 0.003, g = 0.74), and mindfulness measured by BCT was marginally significant (F = 3.6, *p* = 0.060, g = 0.45). Overall, these results support the efficacy of the shortened programme.

### 3.3. Effects of Bringing the Programme into a Natural Setting

Results concerning the potential differences between the two mindfulness groups in comparison with the control group can be found in Table 4. Means and standard deviations for both the primary and secondary outcomes before and after treatment and at the 3-month follow-up are shown in Table 5. Concerning the primary outcomes, an interaction analysis including all three groups showed no interaction effect on perceived stress post-treatment (F = 2.0, *p* = 0.146, g = 0.36). Neither the indoor mindfulness treatment nor the mindfulness treatment in a natural outdoor setting showed significant results compared with the control group, although the difference between the indoor mindfulness treatment and the control group was of medium effect size, as noted above. At follow-up, the interaction term concerning perceived stress was non-significant (F = 1.0, *p* = 0.373, g = 0.18). Concerning self-compassion, no overall between-group effects were observed post-treatment (F = 0.6, *p* = 0.532, g = 0.20). However, there was a trend-wise significant interaction at follow-up (F = 2.9, *p* = 0.058, g = 0.31). When we explored this finding, both the indoor mindfulness treatment and the mindfulness treatment in a natural outdoor setting significantly outperformed the control group.

Concerning the secondary outcomes post-treatment, group differences were only explored for BCT (F = 3.6, *p* = 0.032, g = 0.94). Both the indoor mindfulness treatment and the mindfulness treatment in a natural outdoor setting significantly exceeded the control group. At follow-up, group differences were detected for mindfulness only (FFMQ) (F = 4.0, *p* = 0.021, g = 0.37). Both the indoor mindfulness treatment and the mindfulness treatment in a natural outdoor setting significantly exceeded the control group. For CNS, no group differences were detected post-treatment, although a medium effect size was detected when we compared mindfulness in a natural outdoor setting with indoor mindfulness (F = 2.9, *p* = 0.096, g = 0.52). At follow-up, CNS for mindfulness in a natural outdoor setting significantly exceeded the control (F = 5.4, *p* = 0.023, g = 0.52).

## 4. Discussion

The 5-day residential mindfulness programme—conducted both indoors and in a natural outdoor setting—showed significant positive medium-sized effects on self-compassion at the 3-month follow-up compared with a passive control condition. No significant effects on perceived stress levels were found either post-treatment or at follow-up; however, a post-treatment effect of moderate size might be considered clinically relevant.

A medium- to large-sized positive effect on trait mindfulness after a behavioural task was found at the end of the programme for the two intervention groups, and although this measure did not remain significant 3 months later, a self-report measure indicated a lasting small- to medium-sized effect on trait mindfulness at follow-up. The self-report measure Connectedness to Nature was the only outcome measure with an incremental effect in nature for the residential mindfulness programme. These results contribute to the field by suggesting two potentially effective short residential alternatives to mindfulness programmes with weekly attendance.

### 4.1. Effects of the Residential Mindfulness Training Programme

Residential programmes [47,48] and the 8-week MBSR programme [49] have been shown to have a positive effect on stress levels, self-compassion, and trait mindfulness, but neither our study nor previous studies have compared a residential format against the same programme with weekly sessions [18]. However, benchmarking the effects obtained in the present study with those obtained on average in 8-week mindfulness programmes suggests comparable effects. Indeed, our findings suggest that the effects on stress levels of our 5-day indoor residential mindfulness programme (g = 0.56) may be comparable to effects obtained in 8-week MBSR/MBCT programmes (Cohen’s d = 0.32–0.78) [50]. Furthermore, it may be advantageous to offer the programme in an alternative format as in this study, especially when participants are challenged by—or even drop out due to—factors such as weekly attendance or distance to the mindfulness classes. This is supported by a dropout rate of only 3% in this RCT compared with dropout levels of 16–29% reported by Khoury [14] and Nam et al. [15].

### 4.2. Mindfulness in the Indoor Setting Compared with the Natural Outdoor Setting

When the programme in the indoor and natural outdoor settings was compared, the effects appeared to be equal. In contrast to recent research [27], we did not find an enhanced effect of mindfulness when the programme was conducted in the natural outdoor setting, except on CNS. An explanation may be that both of the settings used in our study were restorative. According to ART, human-made indoor environments may also have the capacity to restore attention [22]. The indoor environment of our study was designed with an emphasis on cohesion, scope, and expansive natural light from above [51], and it may have provided qualities that would be considered restorative according to ART.

The present mindfulness programme is designed to be carried out in an indoor setting, and this may in part explain the lack of incremental effects in the natural outdoor setting. The programme mainly consists of inward-focused attention during meditations that aim to create a robust capacity for the mind to be calm and stable so that stimuli do not easily trigger wandering thoughts [52]. This approach may reduce receptivity to the benefits of the natural environment compared with a more outward-oriented or open-awareness meditation. It may be that the sensory processes stimulated by nature, with the capacity to positively activate the parasympathetic nervous system [53,54], are lesser stimulated with inward-focused attention during meditation, and hence that the capacity of natural settings to restore directed attention may be reduced.

### 4.3. Further Research

A number of adjustments should be considered for scaling up this project into a larger RCT following international guidelines for developing and evaluating interventions. First, in order to avoid contamination, the indoor group should have limited access to outdoor. Second, in order to take more advantage of the potential benefits of the natural environment, a redesigned intervention should test whether a stability of mind can be obtained when one meditates with outward focus points such as sounds or with open awareness as in the MBSR curriculum [6]. This could be achieved either by spending time in a natural restorative setting before meditation [55] to clear participants’ minds and recharge their directed attention [21] or through exposure to nature per se, as suggested by Tang and Posner [56]. Third, directly comparing the retreat format with 8-week programmes is another important step, as is investigating possible moderators such as the specific qualities of the setting.

### 4.4. Limitations

This study was a relatively small pilot study, and although it was statistically powered to detect overall between-group differences of an effect size of d = 0.5, it was not sufficiently powered to detect the smaller effect sizes that we would expect from comparisons between the two intervention groups. However, if interpreted with regard to their effect size, our results can serve as a guide for future and larger studies if the following important limitations are borne in mind. Recruitment proved to be challenging, and an extended recruitment period was necessary due to the slow intake. However, the measurement of perceived stress levels just before the programme started ensured continued eligibility. The extended period from randomisation to the first day of intervention may explain the high dropout level during this phase (35%). Another consequence of the extended recruitment period was the postponement of the control group until year two. As the study participants were aware of the overall research questions, we cannot rule out potential interaction effects between group allocation and participants’ expectations at any of the endpoints. Furthermore, the participants in the indoor group were exposed to an outdoor environment as they slept outdoors, but this environment did not contain the rich qualities of nature that were offered for the outdoor group; thus, any potential confounding effects of nature may be limited. Another potential confounder to many outdoor ‘nature’ studies, that should be considered for future studies, are the various environmental variables (temperature, humidity, noise, safety concerns from being in an open area, etc.) of which one or several may hold the potential to offset the restorative aspects of the natural setting. Lastly, the homogenous sample of Danish students limits the generalisability of the findings.

## 5. Conclusions

The present pilot trial found that a 5-day residential indoor mindfulness intervention, based on the 8-week MBSR programme, positively improved self-compassion among Danish students at follow-up and found a clinically relevant effect size for reduced stress post-treatment. Trait mindfulness was positively affected both post-treatment and at follow-up. The same curriculum taught in a natural outdoor setting had equally positive effects, with incremental effects for connectedness to nature. A larger-scale RCT employing an adjusted programme will shed light on whether this programme format can provide clinically relevant improvements over indoor programmes with weekly attendance.

## Figures and Tables

**Table 1:** Programme for both intervention groups.

Time	Day 1	Day 2	Day 3	Day 4	Day 5	Day 6
7.00–8.00		Sitting, yoga and body scan	Sitting, mindful movement/sitting	Sitting, yoga, sitting	Sitting, yoga, sitting	Sitting, walking, sitting (no guidance)
9.30–12.30		Session 1, second part	Session 3	Sessions 5–6	‘All day’ in silence ends	Session 8, second part (until 11:30 a.m.)
14.30–18.00		Session 2	Sessions 4–5	‘All day’ in silence begins	Session 7	
19.30–20.30	Presentation, silent walk, Session 1, first part	Silent walk, circle, economy of words	Silent walk, circle reflections	Sitting, silent walk, sitting	Sitting, silent walk	

Note. Session numbers, including ‘all day’, refer to the MBSR curriculum described in Table 2. ‘Sitting’ is formal seated meditation. ‘Walk’ refers to formal walking meditation in the morning and informal common mindful walking in the evening.

**Table 2:** Content of sessions in the MBSR curriculum.

Session	Content
1	Introduction to the programme, theoretical underpinnings of mindfulness. Guided reflections and sitting meditation, mindful eating, body scan, and yoga.
2	Guided yoga, sitting meditation, and body scan. Theme: how we perceive the world.
3	Guided yoga, sitting, and walking meditation. Themes: formal versus informal meditation/presence, and awareness of pleasant events.
4	Guided yoga and sitting meditation. Theme: awareness of unpleasant events, focusing on stress reactivity.
5	Guided yoga, sitting meditation. Themes: stress, reacting versus responding to stressful events.
6	Guided yoga, sitting meditation. Theme: communication. Illustrative exercises including different communication styles and behaviour patterns.
All day	Guided yoga, sitting and walking meditation, body scan, eating meditation, mountain or lake meditation, loving–kindness meditation, and visual meditation.
7	Guided yoga and sitting meditation. Inclusion of aspects from previous sessions with emphasis on making the practice one’s own.
8	Guided body scan, yoga, and sitting meditation. Guided reflections on the course and future actions.

Note. Guided meditations for the most part, followed by group discussion to enhance awareness of the meditation experience.

**Table 3:** Group descriptives and comparisons at baseline.

	Group	*N*	Mean (SD)	Range	CI	ANOVA *F* (*p*)
Age, years						0.821 (0.445)
	Indoor	20	31.65 (7.45)	24–54	28.16–35.14	
	Nature	22	31.27 (9.59)	21–60	27.02–35.53	
	Control	18	28.61 (5.91)	22–44	25.67–31.55	
	Total	60	30.60 (7.91)	21–60	28.56–32.64	
Prior mindfulness training						0.2 (0.790)
	Indoor	20	0.32 (0.48)	0–1	0.09–0.55	
	Nature	22	0.24 (0.44)	0–1	0.04–0.44	
	Control	18	0.22 (0.43)	0–1	0.01–0.43	
	Total	60	0.26 (0.44)	0–1	0.14–0.37	
Prior exposure to nature						0.055 (0.947)
	Indoor	20	6.05 (1.79)	3–10	5.21–6.89	
	Nature	22	6.27 (2.35)	2–11	5.23–7.32	
	Control	18	6.17 (2.33)	2–10	5.01–7.33	
	Total	60	6.17 (2.14)	2–11	5.61–6.72	
Outcome variables						
PSS						0.005 (0.995)
	Indoor	20	21.80 (6.49)	5–32	18.76–24.84	
	Nature	22	21.64 (4.77)	14–30	19.52–23.75	
	Control	18	21.78 (5.29)	12–32	19.15–24.41	
	Total	60	21.73 (5.45)	5–32	20.33–23.14	
SCS						0.002 (0.998)
	Indoor	20	34.10 (6.49)	24–48	31.06–37.14	
	Nature	22	33.95 (7.39)	19–52	30.68–37.23	
	Control	18	34.00 (9.29)	17–50	29.38–38.62	
	Total	60	34.02 (7.61)	17–52	32.05–35.98	
FFMQ						0.406 (0.668)
	Indoor	20	118.45 (17.85)	87–151	110.09–126.81	
	Nature	22	120.91 (19.24)	82–161	112.38–129.44	
	Control	18	124.11 (21.06)	84–159	113.64–134.59	
	Total	60	121.05 (19.17)	82–161	116.10–126.00	
BCT						1.1201 (0.309)
	Indoor	20	83.11 (13.36)	50–100	76.86–89.36	
	Nature	22	80.29 (20.56)	22.73–100	71.17–89.40	
	Control	18	88.29 (13.24)	53.85–100	81.70–94.88	
	Total	60	83.63 (16.41)	22.73–100	79.39.87.87	
CNS						0.869 (0.425)
	Indoor	20	52.10 (6.38)	43–64	49.11–55.09	
	Nature	22	48.68 (10.12)	25–63	44.19–53.17	
	Control	18	50.94 (8.60)	40–66	46.67–55.22	
	Total	60	50.50 (8.55)	25–66	48.29–52.71	

Note. Baseline characteristics of intention-to-treat population. SD: Standard deviation. CI: Confidence interval. Prior mindfulness experience: 0 = no prior mindfulness experience, 1 = any mindfulness experience. Prior exposure to nature: 2 = no exposure to nature, 12 = daily exposure to nature, both at home and in public areas. PSS: Perceived Stress Scale. SCS: Self-Compassion Scale. FFMQ: Five-Facet Mindfulness Questionnaire. BCT: Breath-Counting Test. CNS: Connectedness to Nature Scale.

**Table 4:** Results from multilevel interaction analyses.

	Time x Group Interaction Post-Treatment			Time x Group Interaction at Follow-Up		
	*F*	*p*	*g*	*F*	*p*	*g*
**Primary outcome PSS**						
All groups	2.0	0.146	0.36	1.0	0.373	0.18
Control vs. indoor	**3.0**	**0.091**	**0.56**	1.6	0.212	0.29
Control vs. nature	1.2	0.283	0.34	1.2	0.276	0.24
Indoor vs. nature	1.4	0.241	0.36	0.3	0.578	0.12
**Primary outcome SCS**						
All groups	0.6	0.532	0.20	2.9	0.058	0.31
Control vs. indoor	1.0	0.323	0.33	**5.4**	**0.023**	**0.54**
Control vs. nature	1.4	0.242	0.37	**4.9**	**0.030**	**0.49**
Indoor vs. nature	<0.1	0.931	0.03	0.1	0.752	0.07
**Secondary outcome FFMQ**						
All groups	1.1	0.350	0.27	**4.0**	**0.021**	**0.37**
Control vs. indoor	2.3	0.136	0.50	**9.8**	**0.003**	**0.74**
Control vs. nature	1.8	0.187	0.42	**4.8**	**0.032**	**0.48**
Indoor vs. nature	<0.1	0.982	0.01	0.5	0.479	0.15
**Secondary outcome BCT**						
All groups	**3.6**	**0.032**	**0.49**	1.7	0.195	0.24
Control vs. indoor	**7.6**	**0.009**	**0.90**	3.6	0.060	0.45
Control vs. nature	**3.2**	**0.080**	**0.55**	0.9	0.344	0.21
Indoor vs. nature	0.6	0.448	0.23	0.8	0.375	0.20
**Secondary outcome CNS**						
All groups	2.1	0.135	0.37	2.4	0.096	0.29
Control vs. indoor	0.2	0.676	0.15	1.6	0.210	0.30
Control vs. nature	2.2	0.150	0.46	**5.4**	**0.023**	**0.52**
Indoor vs. nature	**2.9**	**0.096**	**0.53**	0.7	0.399	0.18

Note. Significant results and results with effect size of g ≥ 0.5 in bold type. PSS: Perceived Stress Scale. SCS: Self-Compassion Scale. FFMQ: Five-Facet Mindfulness Questionnaire. BCT: Breath-Counting Test. CNS: Connectedness to Nature Scale.

**Table 5:** Means and standard deviations for all outcomes pre-intervention, post-intervention, and at 3-month follow-up.

		n	PSS	SCS	FFMQ	BCT	CNS
Pre	Nature	22	21.6 (4.8)	34.0 (7.4)	120.9 (12.2)	80.3 (13.4)	48.7 (10.1)
	Indoor	20	21.8 (6.5)	34.1 (6.4)	118.5 (17.8)	83.1 (13.3)	52.1 (6.4)
	Control	18	21.8 (5.3)	34.0 (9.3)	124.1 (21.1)	88.3 (13.22)	50.5 (8.5)
Post	Nature	22	21.6 (4.9)	36.1 (9.1)	128.7 (21.1)	87.6 (8.8)	51.5 (9.5)
	Indoor	18	19.4 (7.7)	36.7 (10.5)	125.7 (20.7)	94.1 (9.4)	51.5 (9.5)
	Control	18	22.9 (6.7)	33.8 (10.7)	124.4 (25.4)	84.8 (18.8)	51.7 (8.2)
FU	Nature	21	31.2 (3.6)	39.8 (7.8)	132.5 (21.2)	84.3 (13.1)	52.5 (10.2)
	Indoor	17	30.1 (3.8)	41.1 (6.9)	133.8 (20.3)	90.7 (9.5)	54.1 (8.4)
	Control	17	33.5 (3.5)	35.5 (8.9)	125.9 (24.5)	86.5 (15.7)	52.3 (8.0)

Note. Pre: pre-intervention. Post: post-intervention. FU: follow-up. PSS: Perceived Stress Scale. SCS: Self-Compassion Scale. FFMQ: Five-Facet Mindfulness Questionnaire. BCT: Breath-Counting Test. CNS: Connectedness to Nature Scale.

## Data Availability

Data can be obtained from mia@psy.au.dk.
